# Supplementary material for: Single-cell analyses reveal transient retinal progenitor cells in the ciliary margin of developing human retina
Source: Nat Commun. 2024 Apr 26;15:3567. doi: 10.1038/s41467-024-47933-x (PMC11053058; doi:10.1038/s41467-024-47933-x)
Supplement: Supplementary file 1 — Supplementary Information [file 41467_2024_47933_MOESM1_ESM.pdf]

Single-cell analyses reveal transient retinal progenitor cells in the ciliary margin of developing human retina

Birthe Dorgau [1*], Joseph Collin [1*], Agata Rozanska [1], Darin Zerti [1,2], Adrienne Unsworth [1], Moira Crosier [1], Rafiqul Hussain [1], Jonathan Coxhead [1], Tamil Dhanaseelan [1], Aara Patel [3], Jane C Sowden [3], David R FitzPatrick [4], Rachel Queen [1#] and Majlinda Lako [1#]

* Both authors contributed equally

Senior corresponding authors

* Corresponding authors. Email: majlinda.lako@ncl.ac.uk and rachel.queen@ncl.ac.uk

This PDF file includes:

Supplementary Figures S1 to S11

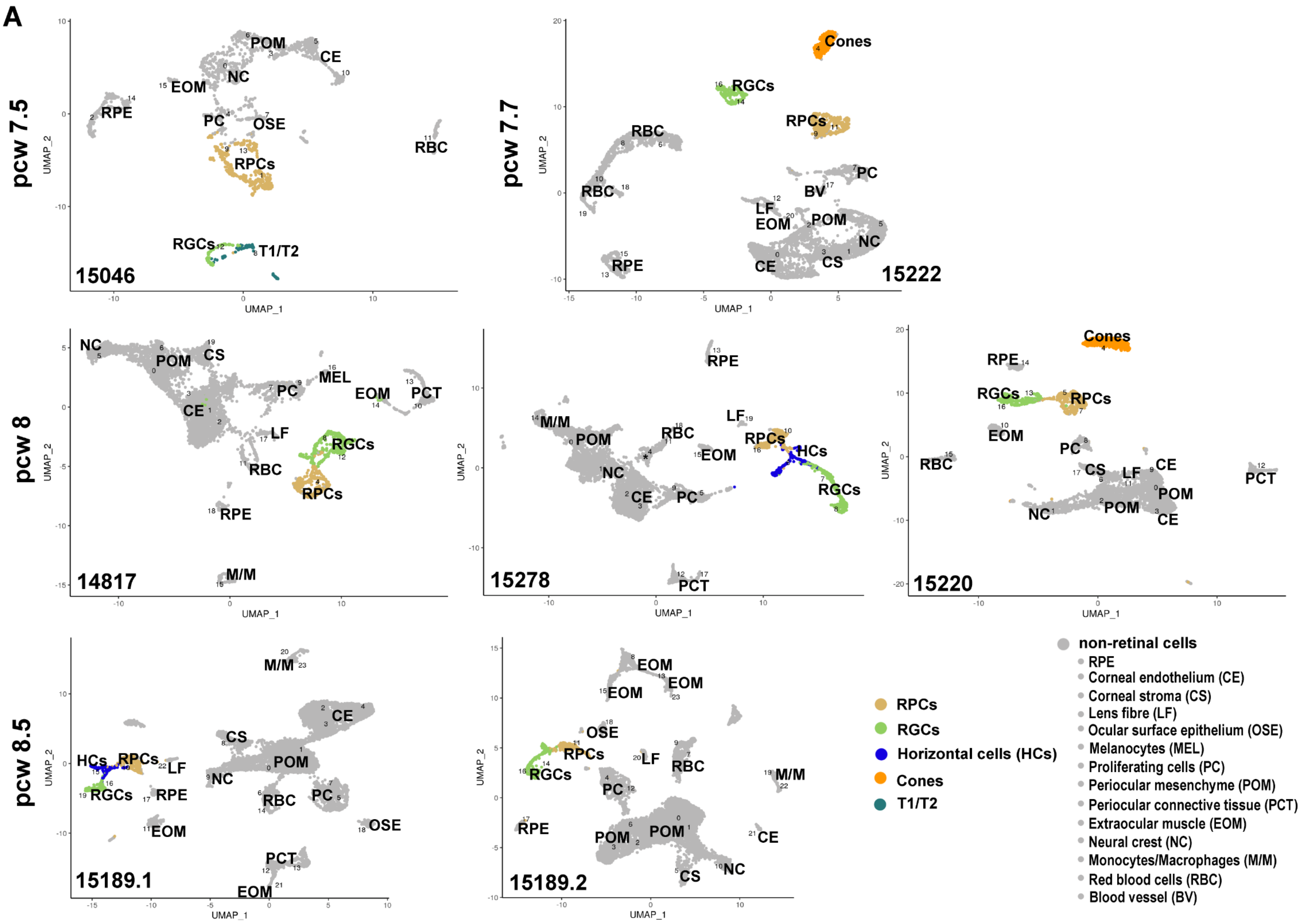
A
pcw 7.5
15046
POM
CE
NC
EOM
RPE
PC
OSE
RBC
RPCs
RGCs
T1/T2
pcw 7.7
15222
Cones
RGCs
RPCs
RBC
PC
BV
LF
EOM
POM
NC
CS
CE
RPE
pcw 8
14817
NC
POM
CS
MEL
PC
EOM
PCT
CE
LF
RGCs
RBC
RPCs
RPE
M/M
15278
RPE
M/M
POM
RBC
LF
RPCs
HCs
EOM
NC
CE
PC
RGCs
PCT
15220
Cones
RPE
RGCs
RPCs
EOM
RBC
PC
CS
LF
CE
PCT
POM
NC
pcw 8.5
15189.1
M/M
CE
CS
HCs
RPCs
LF
POM
NC
RGCs
RPE
RBC
PC
OSE
EOM
PCT
15189.2
EOM
OSE
RPCs
RGCs
LF
RBC
M/M
PC
RPE
POM
CE
CS
NC
UMAP_1
UMAP_2
RPCs
RGCs
Horizontal cells (HCs)
Cones
T1/T2
non-retinal cells
RPE
Corneal endothelium (CE)
Corneal stroma (CS)
Lens fibre (LF)
Ocular surface epithelium (OSE)
Melanocytes (MEL)
Proliferating cells (PC)
Periocular mesenchyme (POM)
Periocular connective tissue (PCT)
Extraocular muscle (EOM)
Neural crest (NC)
Monocytes/Macrophages (M/M)
Red blood cells (RBC)
Blood vessel (BV)

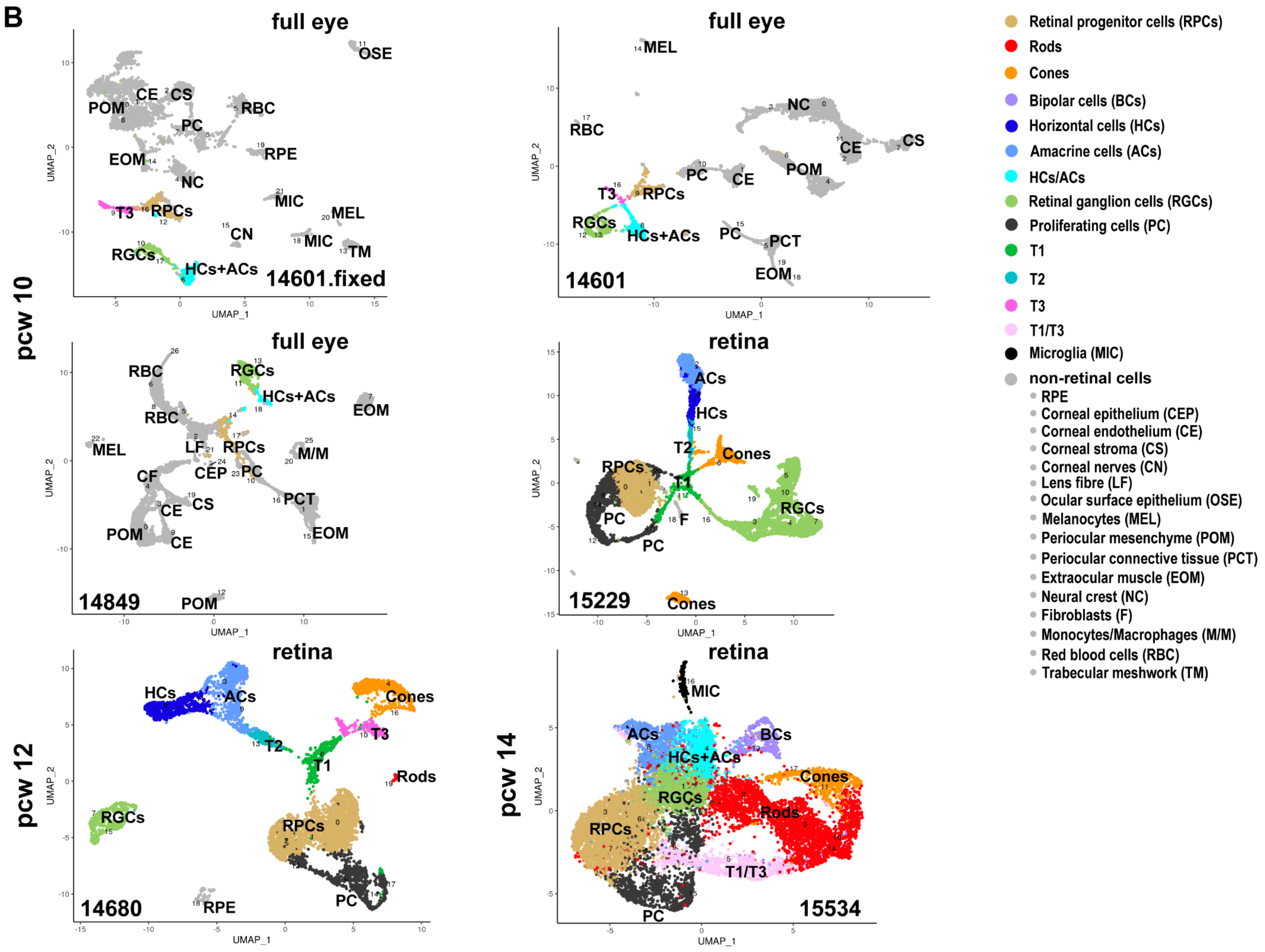
B
pcw 10
pcw 12
pcw 14
full eye
14601.fixed
full eye
14601
full eye
14849
retina
15229
retina
14680
retina
15534
OSE
CE
CS
POM
RBC
PC
RPE
EOM
NC
MIC
MEL
T3
RPCs
CN
TM
RGCs
HCs+ACs
M/M
LF
CEP
CF
PCT
ACs
HCs
T2
Cones
T1
F
Rods
BCs
T1/T3
UMAP_1
UMAP_2
Retinal progenitor cells (RPCs)
Rods
Cones
Bipolar cells (BCs)
Horizontal cells (HCs)
Amacrine cells (ACs)
HCs/ACs
Retinal ganglion cells (RGCs)
Proliferating cells (PC)
T1
T2
T3
T1/T3
Microglia (MIC)
non-retinal cells
RPE
Corneal epithelium (CEP)
Corneal endothelium (CE)
Corneal stroma (CS)
Corneal nerves (CN)
Lens fibre (LF)
Ocular surface epithelium (OSE)
Melanocytes (MEL)
Periocular mesenchyme (POM)
Periocular connective tissue (PCT)
Extraocular muscle (EOM)
Neural crest (NC)
Fibroblasts (F)
Monocytes/Macrophages (M/M)
Red blood cells (RBC)
Trabecular meshwork (TM)

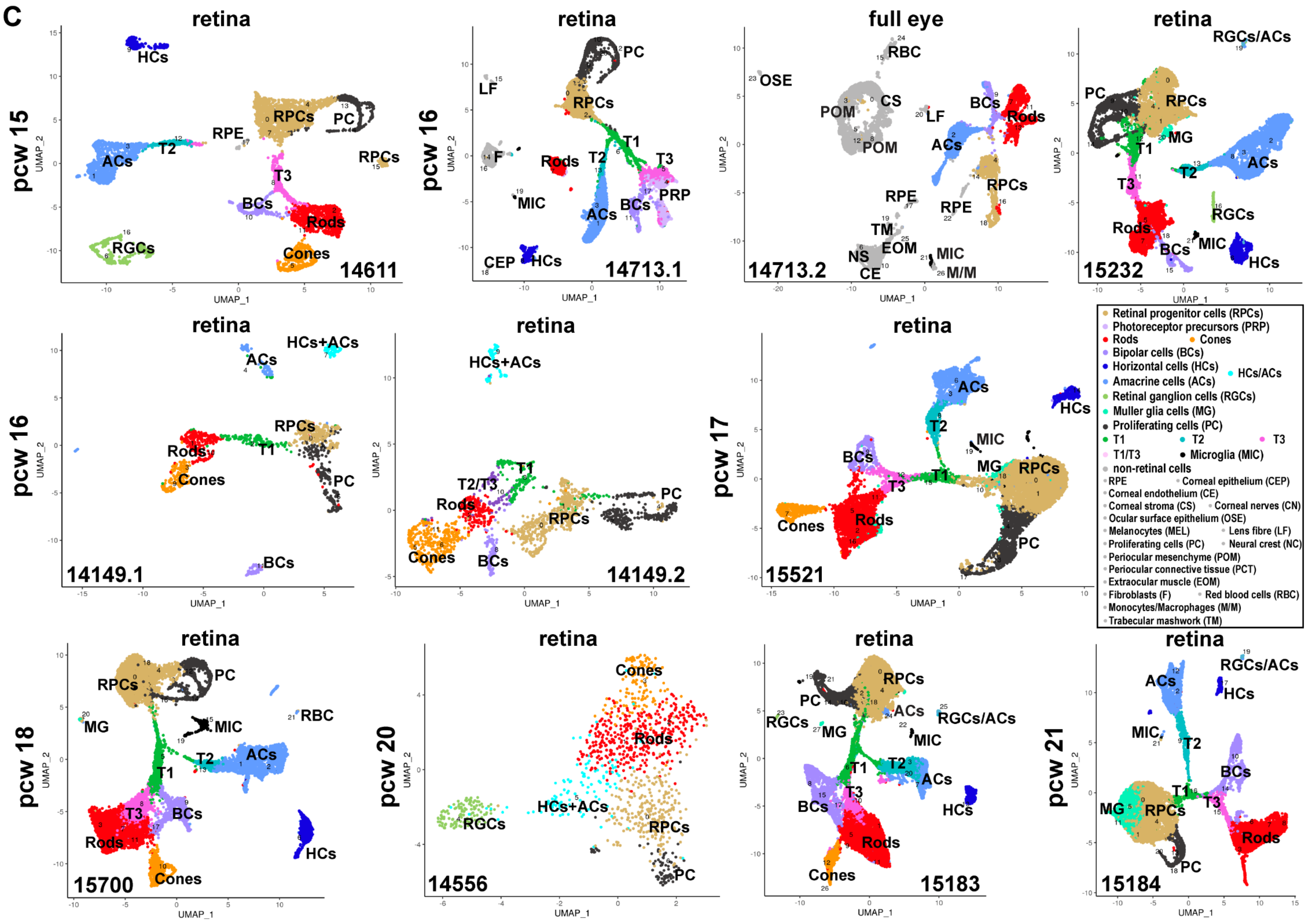
C
retina
full eye
pcw 15
pcw 16
pcw 17
pcw 18
pcw 20
pcw 21
14611
14713.1
14713.2
15232
14149.1
14149.2
15521
15700
14556
15183
15184
UMAP_1
UMAP_2
HCs
RPCs
PC
RPE
ACs
T2
T3
BCs
Rods
RGCs
Cones
LF
F
MIC
CEP
T1
PRP
OSE
RBC
CS
POM
TM
EOM
NS
CE
M/M
RGCs/ACs
MG
HCs+ACs
T2/T3
Retinal progenitor cells (RPCs)
Photoreceptor precursors (PRP)
Rods
Cones
Bipolar cells (BCs)
Horizontal cells (HCs)
HCs/ACs
Amacrine cells (ACs)
Retinal ganglion cells (RGCs)
Muller glia cells (MG)
Proliferating cells (PC)
T1
T2
T3
T1/T3
Microglia (MIC)
non-retinal cells
RPE
Corneal epithelium (CEP)
Corneal endothelium (CE)
Corneal stroma (CS)
Corneal nerves (CN)
Ocular surface epithelium (OSE)
Melanocytes (MEL)
Lens fibre (LF)
Proliferating cells (PC)
Neural crest (NC)
Periocular mesenchyme (POM)
Periocular connective tissue (PCT)
Extraocular muscle (EOM)
Fibroblasts (F)
Red blood cells (RBC)
Monocytes/Macrophages (M/M)
Trabecular mashwork (TM)

Figures S1: Ordered emergence of all retinal cell types during human retinal development. A-C) UMAP of scRNA-Seq from 7.5 – 8. PCW whole eyes (**A**), 10 – 14 PCW whole eyes/isolated foetal retina (**B**) and 15 – 21 PCW whole eyes/isolated foetal retina (**C**). Cluster identity was defined on the basis on expression of retinal specific cell markers shown in Supplementary Data 10. Highly expressed markers for each cluster are shown in Supplementary Data 1. Human developmental biology resource (HDBR) sample accession number is shown on each UMAP together with the embryonic or foetal age.

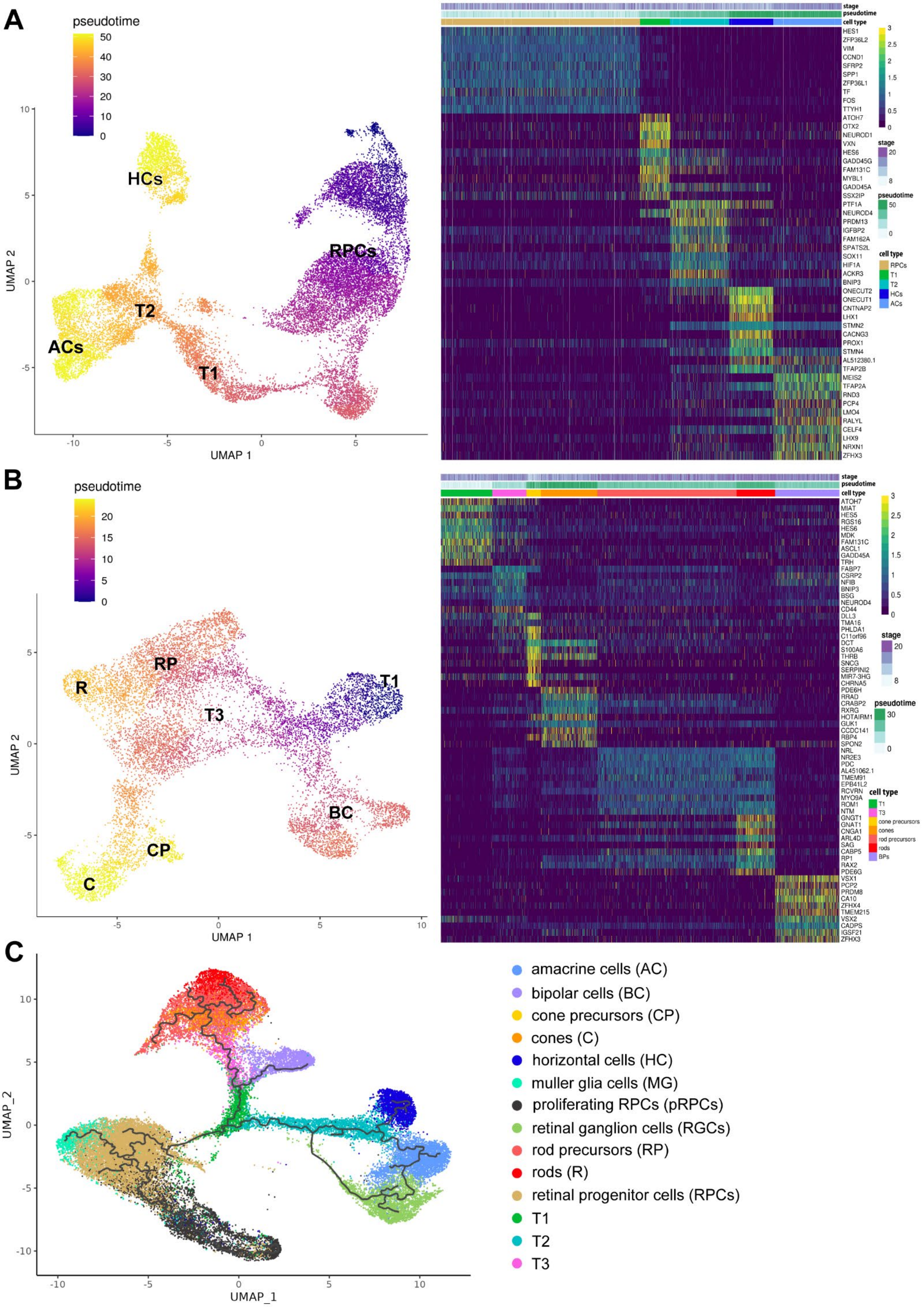
A
pseudotime
50
40
30
20
10
0
HCs
RPCs
T2
ACs
T1
UMAP 2
UMAP 1
stage
pseudotime
cell type
HES1
ZFP36L2
VIM
CCND1
SFRP2
SPP1
ZFP36L1
TF
FOS
TTYH1
ATOH7
OTX2
NEUROD1
VXN
HES6
GADD45G
FAM131C
MYBL1
GADD45A
SSX2IP
PTF1A
NEUROD4
PRDM13
IGFBP2
FAM162A
SPATS2L
SOX11
HIF1A
ACKR3
BNIP3
ONECUT2
ONECUT1
CNTNAP2
LHX1
STMN2
CACNG3
PROX1
STMN4
AL512380.1
TFAP2B
MEIS2
TFAP2A
RND3
PCP4
LMO4
RALYL
CELF4
LHX9
NRXN1
ZFHX3
RPCs
T1
T2
HCs
ACs
B
pseudotime
R
RP
T3
T1
BC
CP
C
UMAP 2
UMAP 1
ATOH7
MIAT
HES5
RGS16
HES6
MDK
FAM131C
ASCL1
GADD45A
TRH
FABP7
CSRP2
NFIB
BNIP3
BSG
NEUROD4
CD44
DLL3
TMA16
PHLDA1
C11orf96
DCT
S100A6
THRB
SNCG
SERPINI2
MIR7-3HG
CHRNA5
PDE6H
RRAD
CRABP2
RXRG
HOTAIRM1
GUK1
CCDC141
RBP4
SPON2
NRL
NR2E3
PDC
AL451062.1
TMEM91
EPB41L2
RCVRN
MYO9A
ROM1
NTM
GNGT1
GNAT1
CNGA1
ARL4D
SAG
CABP5
RP1
RAX2
PDE6G
VSX1
PCP2
PRDM8
CA10
ZFHX4
TMEM215
VSX2
CADPS
IGSF21
ZFHX3
T1
T3
cone precursors
cones
rod precursors
rods
BPs
C
UMAP_2
UMAP_1
amacrine cells (AC)
bipolar cells (BC)
cone precursors (CP)
cones (C)
horizontal cells (HC)
muller glia cells (MG)
proliferating RPCs (pRPCs)
retinal ganglion cells (RGCs)
rod precursors (RP)
rods (R)
retinal progenitor cells (RPCs)
T1
T2
T3

Figure S2: Pseudotime trajectory analysis showing the emergence of retinal cell types from neurogenic progenitors. **A**) Pseudotime trajectory analysis showing transition from RPCs to T1, T2 which give rise to horizontal and amacrine cells and gene expression heatmap indicating the distinct expression patterns of RPCs, T1, T2 and horizontal and amacrine cells. **B**) Pseudotime trajectory analysis showing transition from T1 to T3 bipotent progenitors which give rise to cone and rod photoreceptor and bipolar cells and gene expression heatmap revealing the distinct expression patterns of T1 and T3 progenitors and photoreceptor and bipolar cells. Note transition of mature cones and rods through cone and rod photoreceptors. Highly expressed markers for each cluster along the pseudotime trajectory are shown in Supplementary Data 2. **C**) Pseudotime trajectory over imposed on the integrated scRNA-Seq UMAP demonstrates that horizontal and amacrine cells go through an RPC-T1-T2 transition, whilst photoreceptor and bipolar cells go through an RPC-T1-T3 transition.

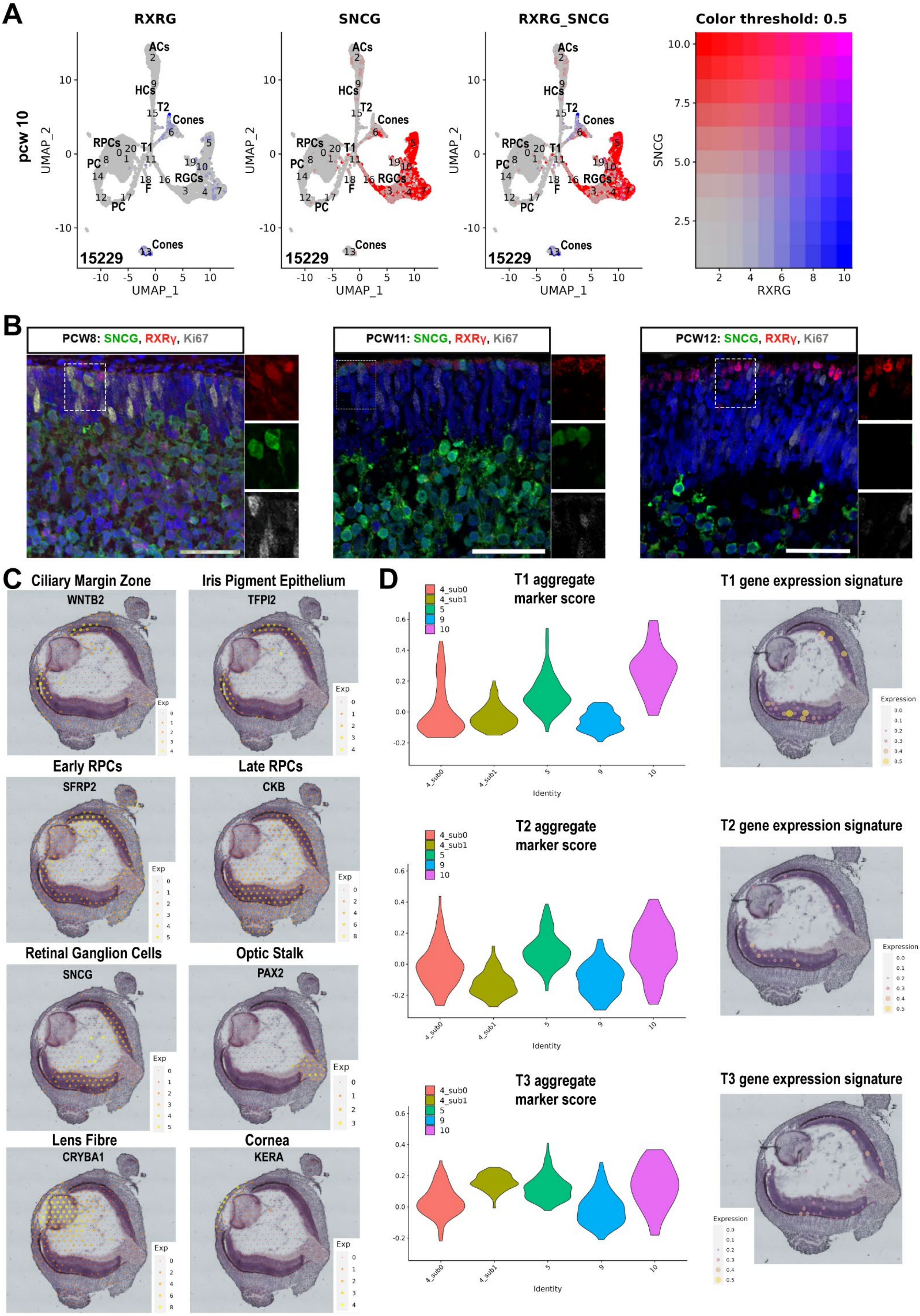
A
RXRG
SNCG
RXRG_SNCG
Color threshold: 0.5
pcw 10
UMAP_2
UMAP_1
ACs
HCs
T2
Cones
RPCs
T1
PC
RGCs
F
15229
SNCG
RXRG
B
PCW8: SNCG, RXRγ, Ki67
PCW11: SNCG, RXRγ, Ki67
PCW12: SNCG, RXRγ, Ki67
C
Ciliary Margin Zone
WNTB2
Iris Pigment Epithelium
TFPI2
Early RPCs
SFRP2
Late RPCs
CKB
Retinal Ganglion Cells
SNCG
Optic Stalk
PAX2
Lens Fibre
CRYBA1
Cornea
KERA
Exp
D
T1 aggregate marker score
T1 gene expression signature
T2 aggregate marker score
T2 gene expression signature
T3 aggregate marker score
T3 gene expression signature
4_sub0
4_sub1
Identity
Expression

Figure S3. Transient expression of RGCs markers in cone photoreceptors during human retinal development and spatial mapping of differentiated and progenitor cells in the developing human eye. A) Overlay expression plots of the scRNA-Seq UMAP of 10 PCPW retina showing expression of the RGC marker SNCG and cone marker RXRG in the RGCs and cone photoreceptor clusters. **B)** Immunofluorescence analysis showing the co-expression of cone photoreceptor marker RXRγ with RGC marker SNCG. Co-expression is strong in the early developmental stages (8 PCW) in both proliferating and non-proliferating cone progenitors but tails off at 12 PCW. Scale bars = 50 µm. **C**) Expression of key markers for various eye and retinal cells superimposed on the ST image of the 8 PCW eye shown in Figure 2A-C. **D**) Expression violin plots of aggregate expression scores showing the predominant spatial expression of T1, T2, and T3 neurogenic progenitors in the outer neuroblastic layer of the central retina in the 8 PCW eye.

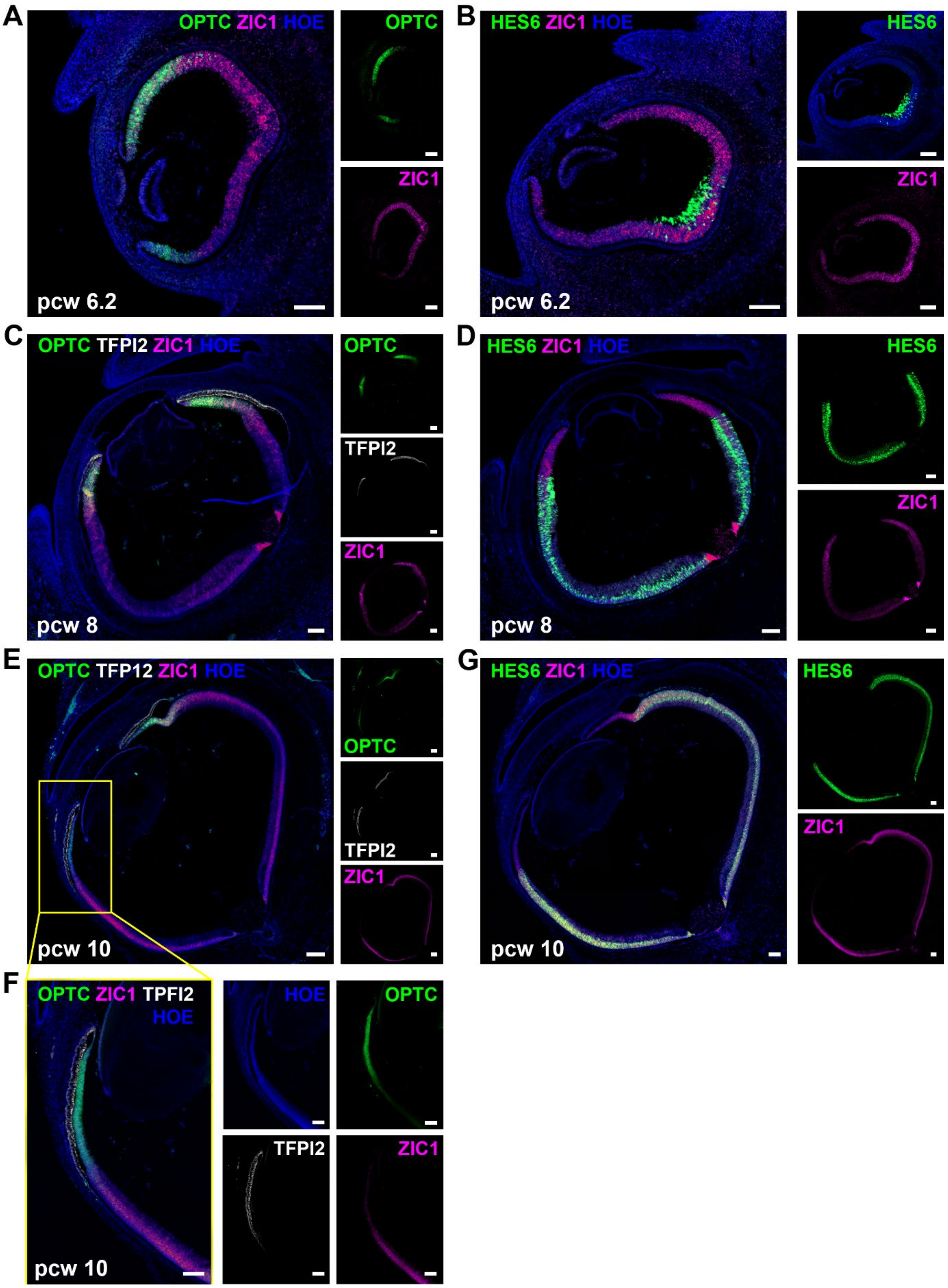

Figure S4: RNAscope reveals strong expression of early RPC marker *ZIC1* in the CMZ of 6.2 -8 PCW human retina. A, C) Overlapping expression of ciliary body marker *OPTC* with early RPC marker *ZIC1* and iris pigmented epithelial marker *TFPI2* in the ciliary margin zone of 8 PCW retina. **B, D, G**) The expression of late RPC/NRPC marker *HES6* is observed in the central retina, but not in the CMZ. **E, F**) Decreasing expression of *ZIC1* in the ciliary margin zone of 10 PCW human retina. Scale bars= 100 µM.

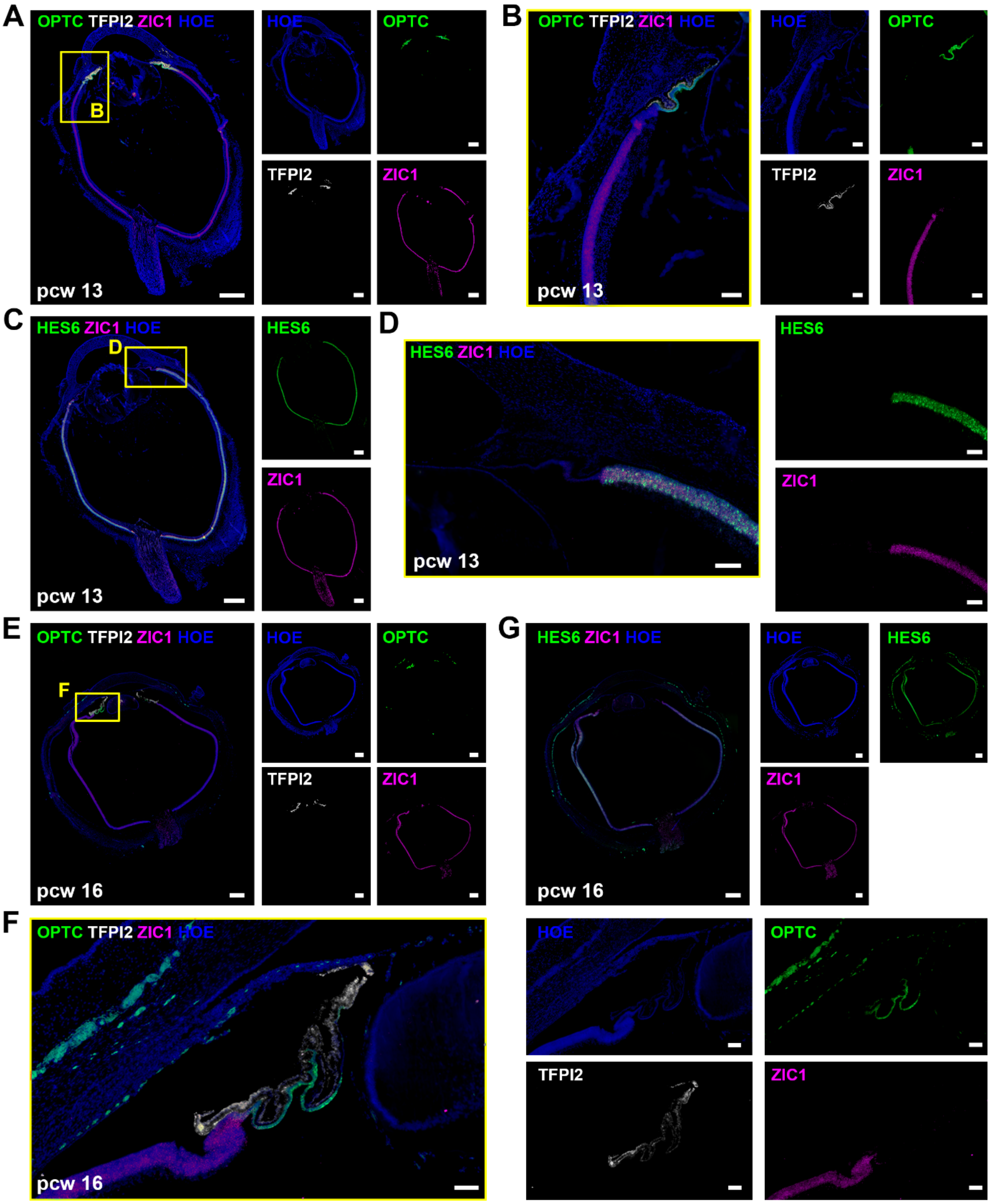

Figure S5: The early RPC marker *ZIC1* is absent from the ciliary margin zone of 13-16 PCW human retina. A, B, E, F) *ZIC1* expression does not co-localise with ciliary body marker *OPTC* and iris pigmented epithelial marker *TFPI2* in the ciliary margin zone of 13-16 PCW. **C, D, G**) Co-localisation of *ZIC1* with *HES6* in the central retina of 13-16 PCW. Scale bars of panel A, C, E and G = 500µm and panels B, E and F (magnifications) = 100µm.

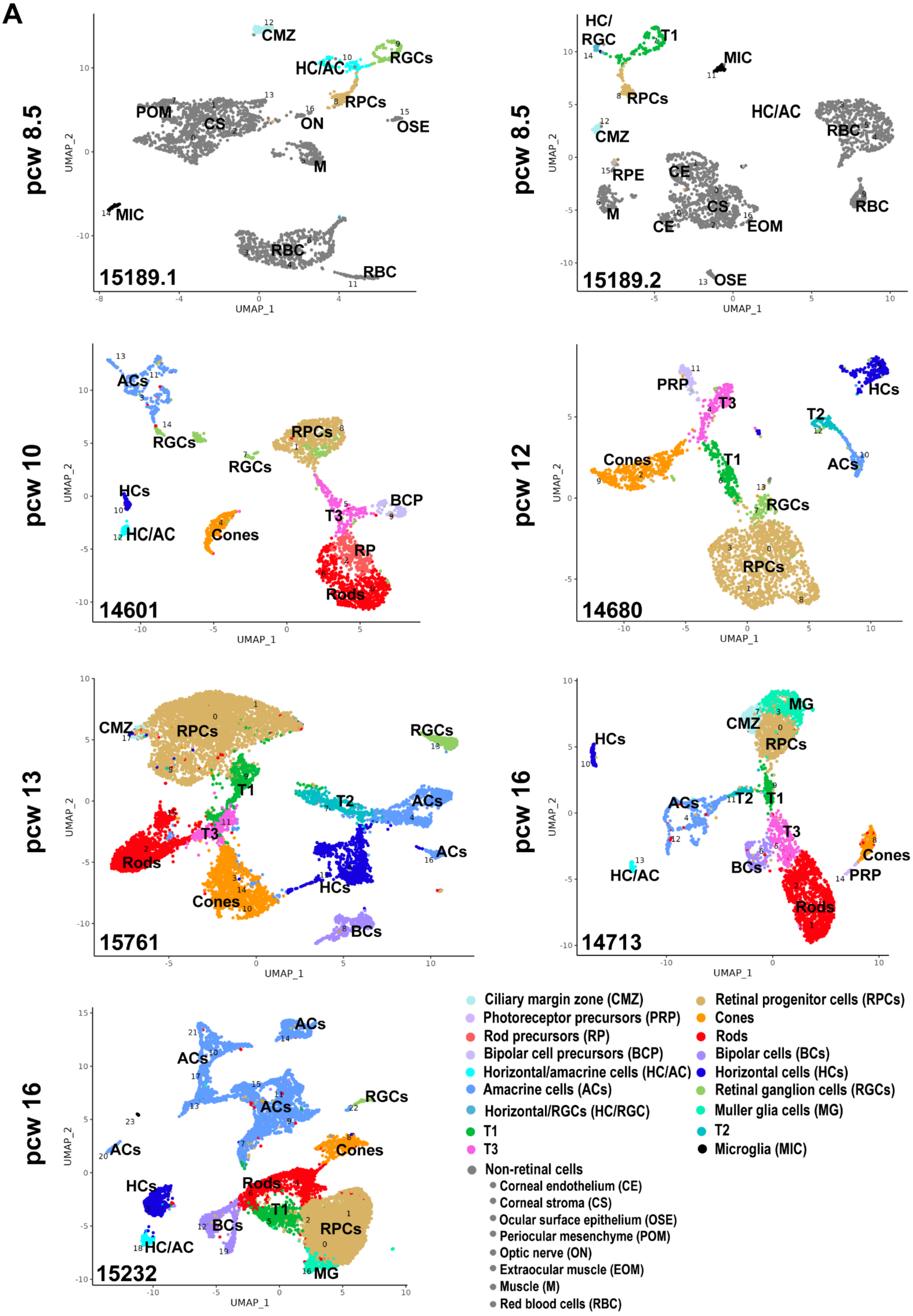

A
pcw 8.5
15189.1
CMZ
HC/AC
RGCs
RPCs
POM
CS
ON
OSE
M
MIC
RBC
RBC
pcw 8.5
15189.2
HC/
RGC
T1
MIC
RPCs
HC/AC
RBC
CMZ
RPE
CE
CS
M
CE
EOM
RBC
OSE
pcw 10
14601
ACs
RGCs
RPCs
RGCs
HCs
BCP
T3
HC/AC
Cones
RP
Rods
pcw 12
14680
PRP
HCs
T3
T2
Cones
T1
ACs
RGCs
RPCs
pcw 13
15761
CMZ
RPCs
RGCs
T1
T2
ACs
Rods
T3
ACs
HCs
Cones
BCs
pcw 16
14713
MG
CMZ
HCs
RPCs
ACs
T2
T1
T3
Cones
HC/AC
BCs
PRP
Rods
pcw 16
15232
ACs
ACs
RGCs
ACs
ACs
Cones
HCs
Rods
T1
BCs
RPCs
HC/AC
MG
UMAP_1
UMAP_2
Ciliary margin zone (CMZ)
Photoreceptor precursors (PRP)
Rod precursors (RP)
Bipolar cell precursors (BCP)
Horizontal/amacrine cells (HC/AC)
Amacrine cells (ACs)
Horizontal/RGCs (HC/RGC)
T1
T3
Non-retinal cells
Corneal endothelium (CE)
Corneal stroma (CS)
Ocular surface epithelium (OSE)
Periocular mesenchyme (POM)
Optic nerve (ON)
Extraocular muscle (EOM)
Muscle (M)
Red blood cells (RBC)
Retinal progenitor cells (RPCs)
Cones
Rods
Bipolar cells (BCs)
Horizontal cells (HCs)
Retinal ganglion cells (RGCs)
Muller glia cells (MG)
T2
Microglia (MIC)

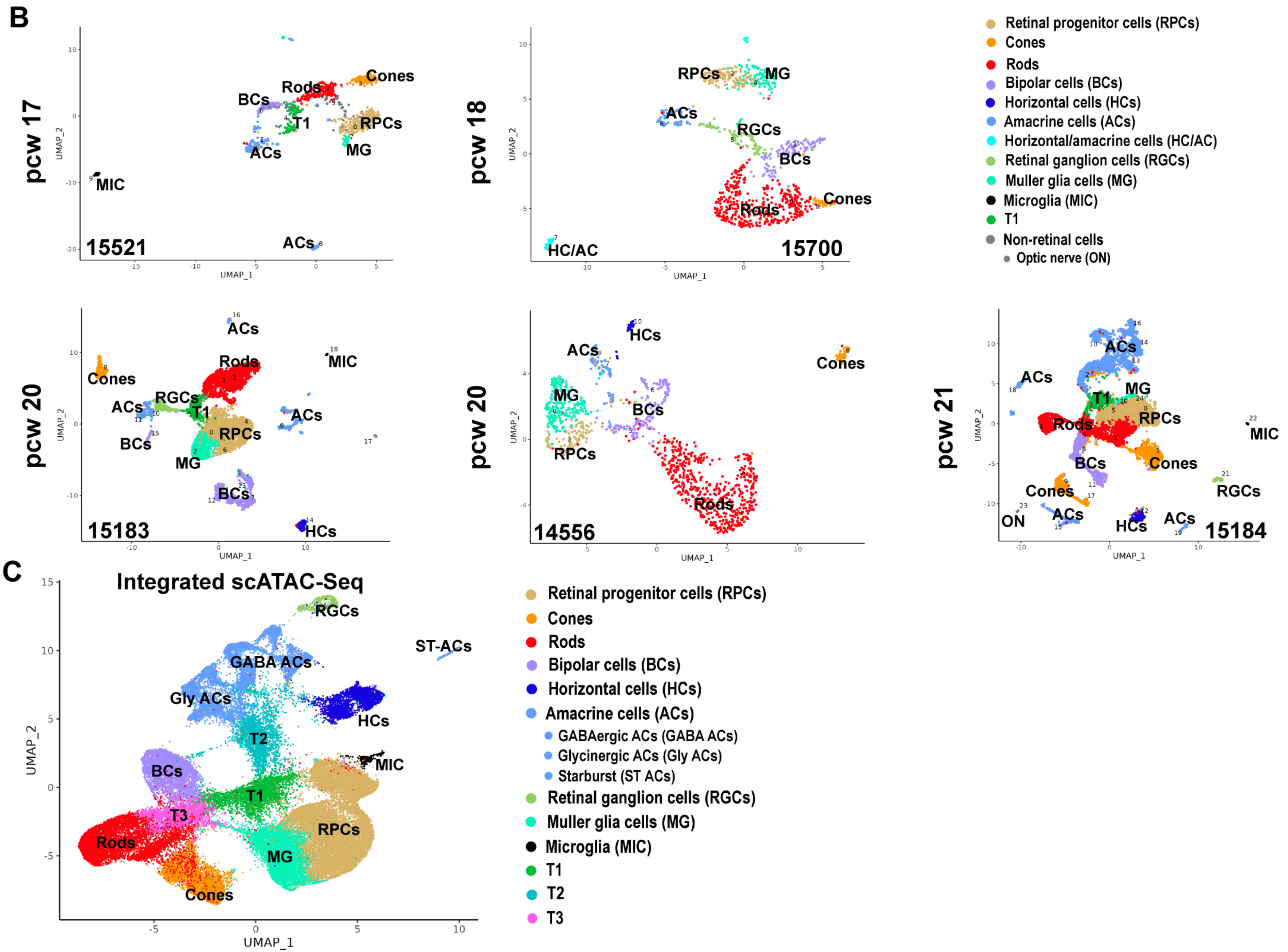
B
pcw 17
15521
Cones
Rods
BCs
T1
RPCs
ACs
MG
MIC
ACs
pcw 18
15700
RPCs
MG
ACs
RGCs
BCs
Cones
Rods
HC/AC
Retinal progenitor cells (RPCs)
Cones
Rods
Bipolar cells (BCs)
Horizontal cells (HCs)
Amacrine cells (ACs)
Horizontal/amacrine cells (HC/AC)
Retinal ganglion cells (RGCs)
Muller glia cells (MG)
Microglia (MIC)
T1
Non-retinal cells
Optic nerve (ON)
pcw 20
15183
ACs
Rods
MIC
Cones
RGCs
ACs
T1
ACs
RPCs
BCs
MG
BCs
HCs
pcw 20
14556
HCs
ACs
Cones
MG
BCs
RPCs
Rods
pcw 21
15184
ACs
ACs
MG
T1
RPCs
Rods
MIC
BCs
Cones
Cones
RGCs
ON
ACs
HCs
ACs
UMAP_1
UMAP_2
C
Integrated scATAC-Seq
RGCs
ST-ACs
GABA ACs
Gly ACs
HCs
T2
BCs
MIC
T1
T3
RPCs
Rods
MG
Cones
Retinal progenitor cells (RPCs)
Cones
Rods
Bipolar cells (BCs)
Horizontal cells (HCs)
Amacrine cells (ACs)
GABAergic ACs (GABA ACs)
Glycinergic ACs (Gly ACs)
Starburst (ST ACs)
Retinal ganglion cells (RGCs)
Muller glia cells (MG)
Microglia (MIC)
T1
T2
T3

Figure S6: Single cell ATAC-Seq of whole human eyes or developing retina. A-B) UMAPs of individual datasets from 8 – 21 PCW. Human developmental biology resource (HDBR) sample accession number are shown on each UMAP together with the embryonic or foetal age. **C**) Integrated scATAC-Seq data of 8-21 PCW. Cluster identity was defined on the basis of gene activity scores, calculated based on open regions of the chromatin of retinal specific cell markers (Supplementary Data 5).

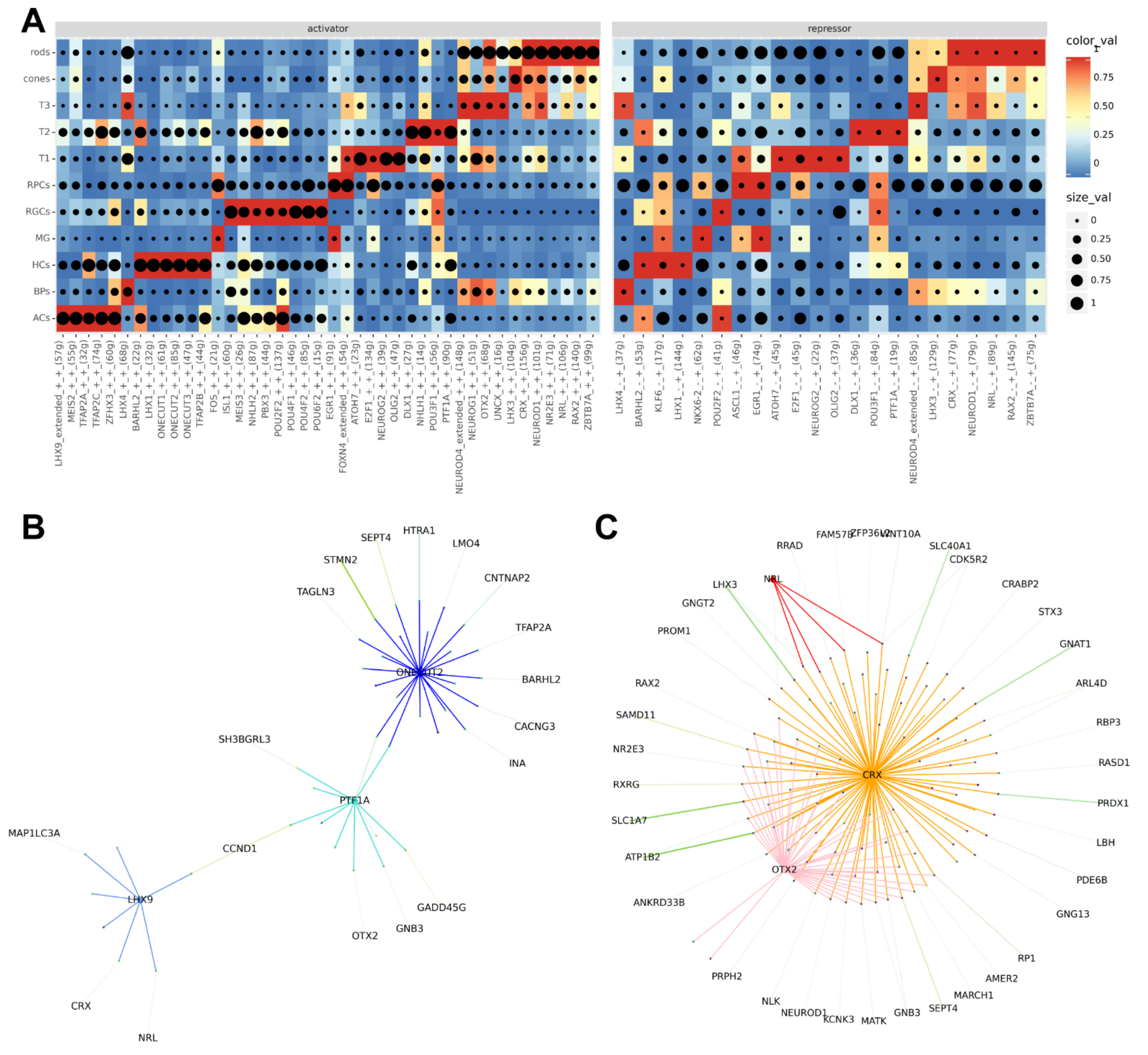

Figure S7: Results of SCENIC+ analysis. A) Heatmap illustrating the expression of cell-type-specific transcription factors (TFs) within the eRegulon. The colours indicate the intensity of expression, and the size of the dot indicates the cell-type specificity (RSS). The cell types are arranged by similarities in gene expression. **B, C**) Enhancer-driven Gene Regulatory Network (eGRN) are depicted for LHX9, PTF1A and ONECUT2 (B) and OTX2, CRX and NRL (C). The TFs are named in the networks and connected to candidate enhancer regions (represented by unlabelled dots) with edges with the following colours: Light blue - LHX9, Turquoise- PTF1A, Dark Blue- ONECUT2, Pink- OTX2, Orange- CRX and Red- NRL. These candidate enhancer regions are subsequently linked to genes (labelled) within the regulon. The edge colour between the candidate enhancer regions and gene is determined by the region-to-gene correlation score.

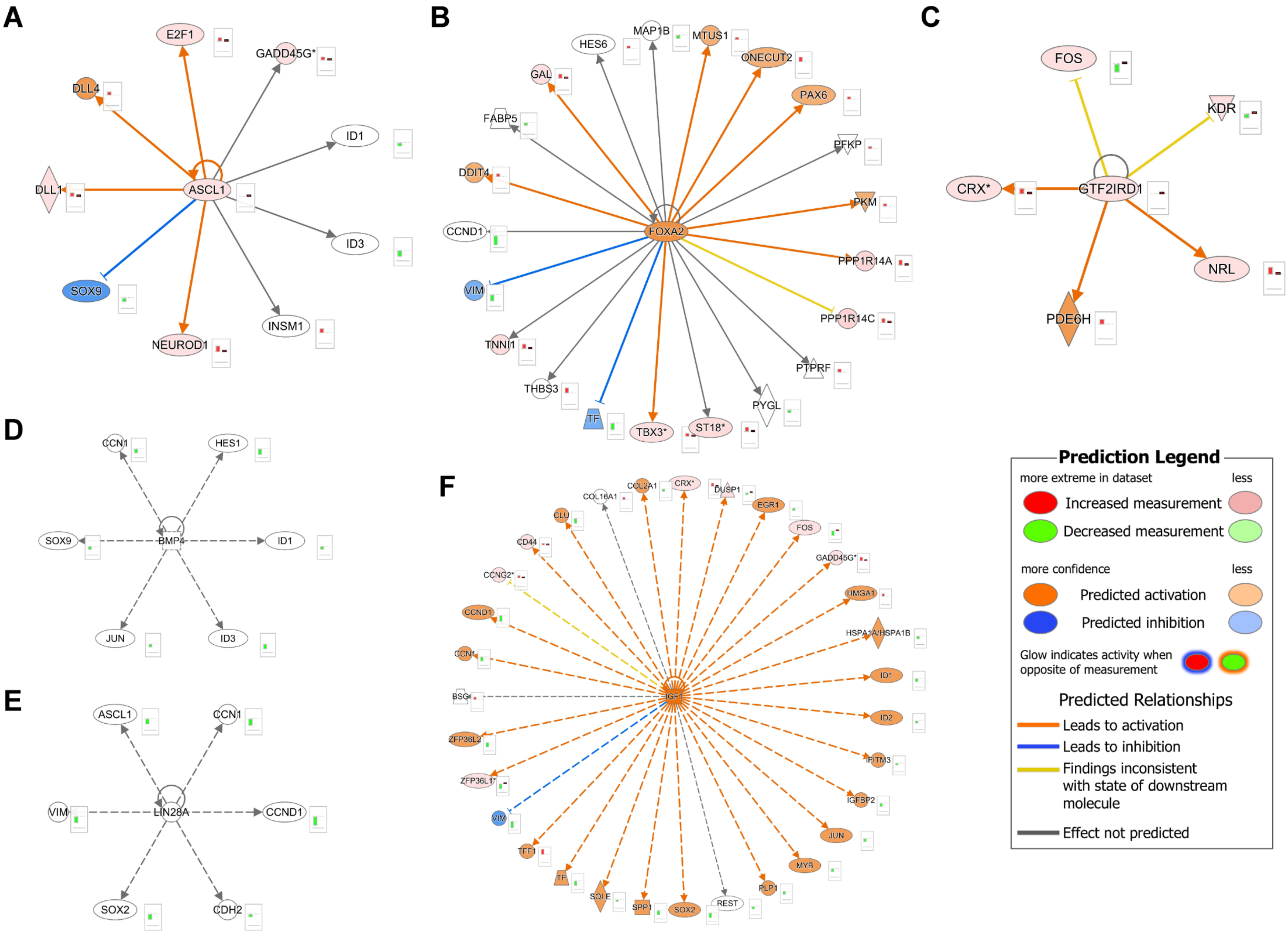
A
E2F1
GADD45G*
DLL4
ID1
DLL1
ASCL1
ID3
SOX9
INSM1
NEUROD1
B
MAP1B
HES6
MTUS1
ONECUT2
GAL
PAX6
FABP5
PFKP
DDIT4
PKM
CCND1
FOXA2
PPP1R14A
VIM
PPP1R14C
TNNI1
PTPRF
THBS3
PYGL
TF
TBX3*
ST18*
C
FOS
KDR
CRX*
GTF2IRD1
NRL
PDE6H
D
CCN1
HES1
SOX9
BMP4
ID1
JUN
ID3
E
ASCL1
CCN1
VIM
LIN28A
CCND1
SOX2
CDH2
F
COL2A1
CRX*
COL16A1
DUSP1
EGR1
CLU
FOS
CD44
GADD45G*
CCNG2*
HMGA1
CCND1
HSPA1A/HSPA1B
CCN1
ID1
BSG
IGF1
ID2
ZFP36L2
IFITM3
ZFP36L1*
IGFBP2
VIM
JUN
TFF1
MYB
TF
PLP1
SQLE
SPP1
SOX2
REST
Prediction Legend
more extreme in dataset
less
Increased measurement
Decreased measurement
more confidence
less
Predicted activation
Predicted inhibition
Glow indicates activity when opposite of measurement
Predicted Relationships
Leads to activation
Leads to inhibition
Findings inconsistent with state of downstream molecule
Effect not predicted

Figure S8: Representative gene regulatory networks in T1 (A, D), T2 (B, E), and T3 (C, F) transient neurogenic progenitors depicting activated and inhibited upstream regulators and their target genes. Upstream regulatory networks were generated with IPA using differentially expressed genes from the scRNA-Seq data and differential accessibility analysis in the scATAC-Seq data. The networks show predictions of upstream regulators which might be activated or inhibited to explain observed upregulation/downregulations in the data. The barplots next to each molecule represent the relative expression in the sRNA-Seq (column 1) and scATAC-Seq datasets (column 2). The colours for the network nodes/barplots indicate observed upregulation/ increased chromatin accessibility (red), predicted upregulation/increased chromatin accessibility (orange), observed downregulation (green) and predicted downregulation/ decreased chromatin accessibility (blue). The colour of the edges represents the relationships between the molecules; orange = prediction and observation are consistent with activation; blue = prediction and observation are consistent with downregulation; yellow = prediction and observation are inconsistent; and grey relationship between the molecules is available in the IPA knowledge database. *- indicates duplicates in scATAC-Seq dataset.

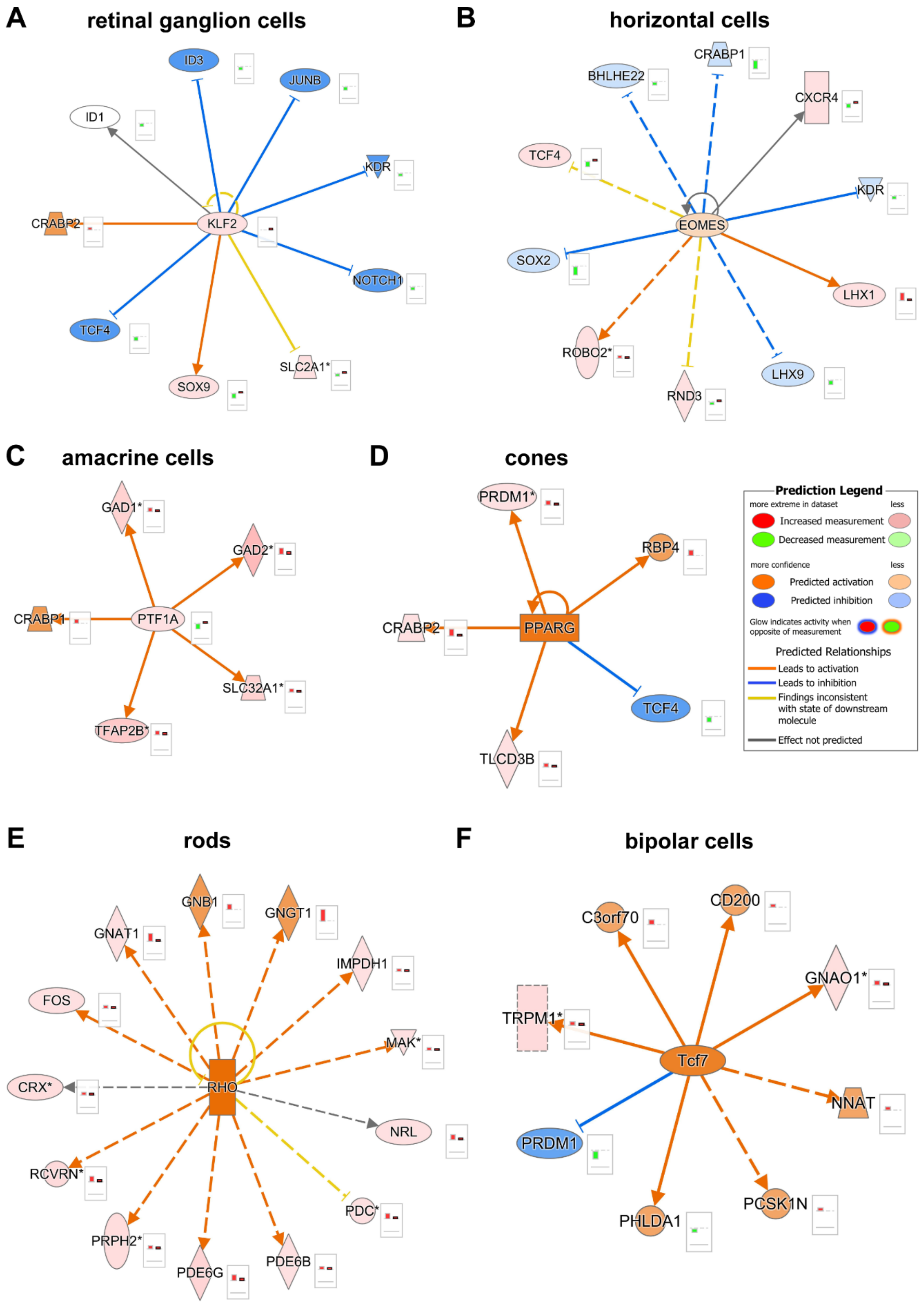

A
retinal ganglion cells
ID3
JUNB
ID1
KDR
CRABP2
KLF2
NOTCH1
TCF4
SLC2A1*
SOX9
B
horizontal cells
CRABP1
BHLHE22
CXCR4
TCF4
KDR
EOMES
SOX2
LHX1
ROBO2*
LHX9
RND3
C
amacrine cells
GAD1*
GAD2*
CRABP1
PTF1A
SLC32A1*
TFAP2B*
D
cones
PRDM1*
RBP4
CRABP2
PPARG
TCF4
TLCD3B
Prediction Legend
more extreme in dataset
less
Increased measurement
Decreased measurement
more confidence
less
Predicted activation
Predicted inhibition
Glow indicates activity when opposite of measurement
Predicted Relationships
Leads to activation
Leads to inhibition
Findings inconsistent with state of downstream molecule
Effect not predicted
E
rods
GNB1
GNGT1
GNAT1
IMPDH1
FOS
MAK*
CRX*
RHO
NRL
RCVRN*
PDC*
PRPH2*
PDE6G
PDE6B
F
bipolar cells
C3orf70
CD200
GNAO1*
TRPM1*
Tcf7
NNAT
PRDM1
PHLDA1
PCSK1N

Figure S9: Representative gene regulatory networks in RGCs (A), horizontal (B), amacrine (C), cones (D), rods (E) and bipolar cells (F). Upstream regulatory networks were generated with IPA using differentially expressed genes from the scRNA-Seq data and differential accessibility analysis in the scATAC-Seq data. The networks show predictions of upstream regulators which might be activated or inhibited to explain observed upregulation/downregulations in the data. The barplots next to each molecule represent the relative expression in the sRNA-Seq (column 1) and scATAC-Seq datasets (column 2). The colours for the network nodes/barplots indicate observed upregulation/ increased chromatin accessibility (red), predicted upregulation/increased chromatin accessibility (orange), observed downregulation (green) and predicted downregulation/ decreased chromatin accessibility (blue). The colour of the edges represents the relationships between the molecules; orange = prediction and observation are consistent with activation; blue = prediction and observation are consistent with downregulation; yellow = prediction and observation are inconsistent; and grey relationship between the molecules is available in the IPA knowledge database. *- indicates duplicates in scATAC-Seq dataset.

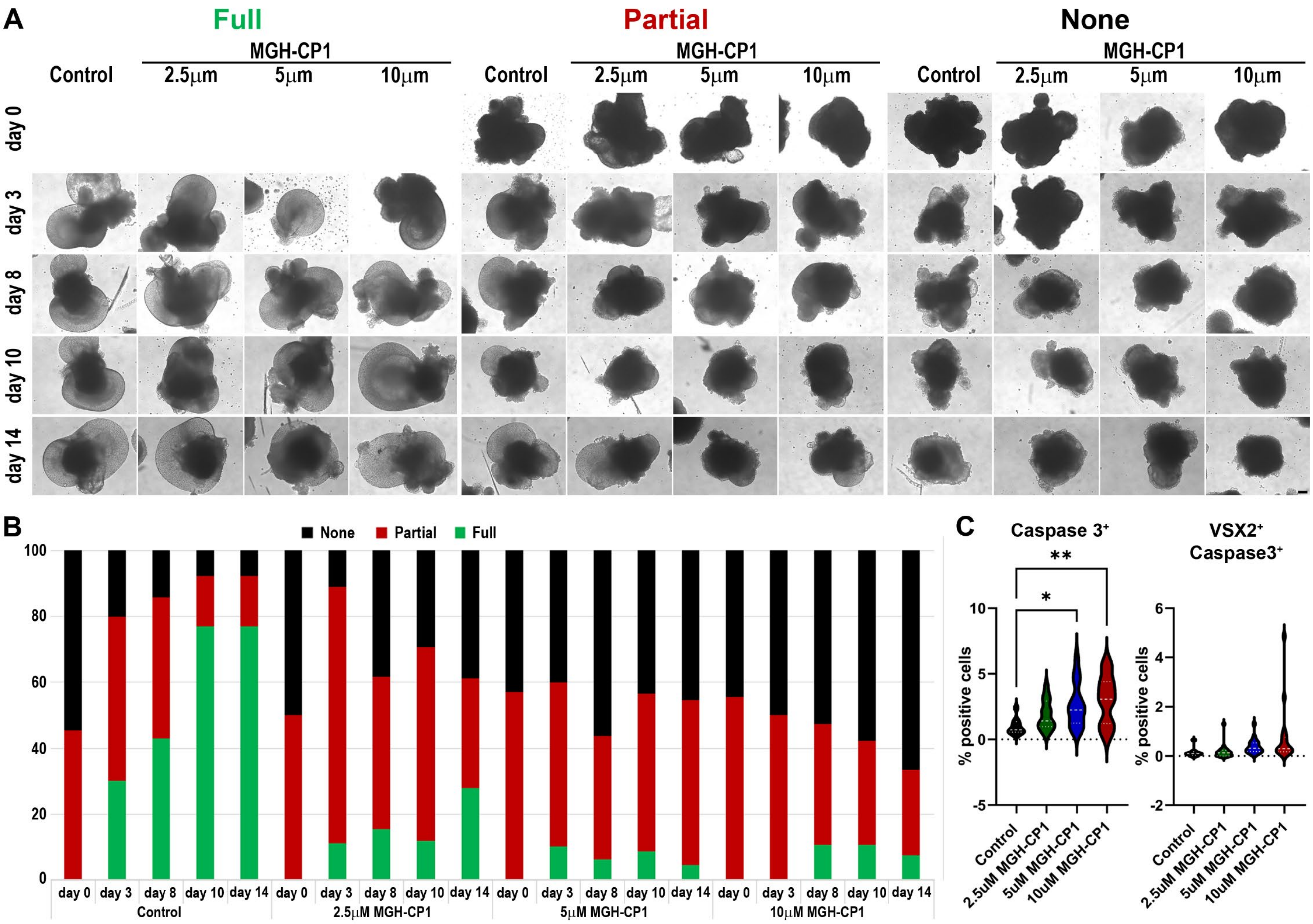
A
Full
Partial
None
MGH-CP1
Control
2.5µm
5µm
10µm
day 0
day 3
day 8
day 10
day 14
B
None
Partial
Full
100
80
60
40
20
0
day 0
day 3
day 8
day 10
day 14
Control
2.5µM MGH-CP1
5µM MGH-CP1
10µM MGH-CP1
C
Caspase 3+
**
*
10
5
0
-5
% positive cells
Control
2.5uM MGH-CP1
5uM MGH-CP1
10uM MGH-CP1
VSX2+ Caspase3+
6
4
2
0
-2

Figure S10. Application of TEAD inhibitor (MGH-CP1) results in loss of retinal lamination. A) Bright field microphotographs showing the presence of retinal organoids with full, partial or no bright phase retinal neuroepithelium at days 0, 3, 8, 10 and 14 post application. Scale bars = 100 µM. **B**) Bar chart showing the percentage of organoids with full, partial or no bright phase retinal neuroepithelium for all the drug concentrations at days 0, 3, 8, 10 and 14 post application. **C**) Violin plot showing a small but significant increase in the percentage of Caspase 3_+ apoptotic cells in retinal organoids treated with 10 µM MGH-CP1. No significant difference across the conditions was observed for $VSX2_+$Caspase 3_+ cells. Data presented as median and quartiles. 9 – 39 retinal organoids per condition were used as shown in the Source Data file. One-way ANOVA with Dunnett's multiple comparisons test (* $p < 0.05$; **$p < 0.01$). Source data are provided as a Source Data file.

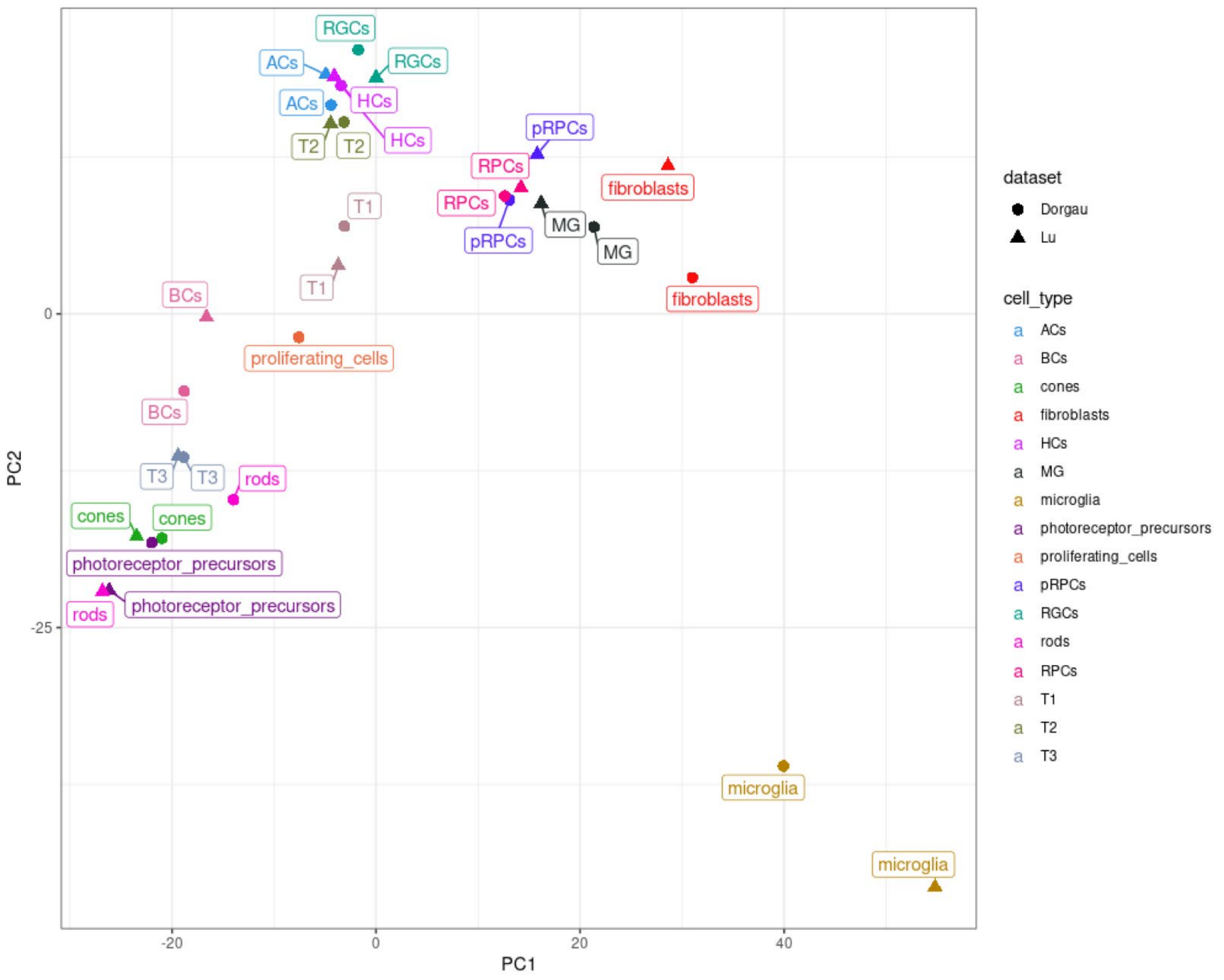

Figure S11. A comparison between scRNA-Seq retinal data generated here and previously published data scRNA-Seq retina data from Lu et al., 2020 [5]. The analysis shows distribution of pseudo-bulk RNA-Seq created from each cell type and each dataset plotted in a reduced-dimensional space based on their principal components. Each point represents a pseudo-bulk sample for a specific cell type, and the distance and direction between points reflect the variance and correlation structure in the original data.
