# Supplementary material for: Single-cell analyses reveal transient retinal progenitor cells in the ciliary margin of developing human retina
Source: Nat Commun. 2024 Apr 26;15:3567. doi: 10.1038/s41467-024-47933-x (PMC11053058; doi:10.1038/s41467-024-47933-x)
Supplement: Supplementary file 3 — Description of Additional Supplementary Files [file 41467_2024_47933_MOESM3_ESM.pdf]

Description of Additional Supplementary Files

Supplementary Data 1: Single cell RNA-Seq analysis of whole human eyes or retina from 7.5-21 PCW. Highly expressed markers for each cell type are shown on separate spreadsheets for each sample together with HDBR sample accession number and dotplots visualising the highly expressed genes for each retinal cell type. A Wilcoxon Rank Sum, two-sided test was used, the adjusted p values corrected for multiple comparisons are shown in the p_val_adj column.

Supplementary Data 2: Single cell RNA-Seq analysis of integrated data set of retinal cells from 7.5- 21 PCW. Highly expressed markers for each cell type in the integrated analysis are shown in the first spreadsheet. Highly expressed markers characterising lineage transitions from RPCs to T1, T2, T3 are shown in the second spreadsheet, those characterising lineage transitions from RPCs to T1, T2 and RGCs, amacrine and horizontal cells are shown in the second and third spreadsheet, and those characterising lineage transitions from RPCs to T1, T2 and amacrine and horizontal cells are shown in the fourth spreadsheet. A Wilcoxon Rank Sum, two-sided test was used, the adjusted p values corrected for multiple comparisons are shown in the p_val_adj column.

Supplementary Data 3: Spatial transcriptomic analysis of 8, 10, 11 and 13 PCW human eyes. Highly expressed markers for each cell type are shown on separate spreadsheets for each sample together with HDBR sample accession number and dotplots visualising the highly expressed genes for each retinal cell type. A Wilcoxon Rank Sum, two-sided test was used, the adjusted p values corrected for multiple comparisons are shown in the p_val_adj column.

Supplementary Data 4: Gene expression signatures of early and late RPCs, and neurogenic T1, T2, and T3 progenitors identified through our own differential gene expression analyses and published literature.

Supplementary Data 5: Single cell ATAC-Seq analysis of whole eyes or retina samples from 8- 21 PCW. Gene activity estimates were generated using Signac. Genes with high activity scores for each cell type are shown on separate spreadsheets for each sample together with HDBR sample accession number. The dotplots show the gene activity score calculated by Signac. This is an estimate of the level of gene expression based on the chromatin accessibility data which is computed by assessing the openness or accessibility of the chromatin around a specific gene locus in individual cells. Higher accessibility in the chromatin region associated with a gene suggests higher activity, and lower accessibility suggests lower activity. The final sheet shows the gene activity scores for the integrated datasets. The retina cell type from each sample were integrated using harmony and the data was then re-clustered to produce these results. A Wilcoxon Rank Sum, two-sided test was used, the adjusted p values corrected for multiple comparisons are shown in the p_val_adj column.

Supplementary Data 6: Differential accessibility peaks for each retinal cell type. Peaks were linked to genes using Cellranger and classified as either promoter, distal or intergenic. The distance from the gene is shown for each peak. Logistic regression logistic regression framework to determine differentially expressed genes. the adjusted p values corrected for multiple comparisons are shown in the p_val_adj column.

Supplementary Data 7: Predicted transcription factor binding for each cell type of the developing human retina. ChromVAR was used to characterise transcription factor motifs for each cell type using a hypergeometric test.

Supplementary Data 8: List of significant regulators of gene expression in RPCs, transient neurogenic progenitors and retinal neurons. A right-tailed Fisher's Exact was used.

Supplementary Data 9: Differentially expressed genes between retinal organoids treated with 10 Um of MGH-CP1 versus those treated with vehicle control (p adjust < 0.05) obtained from bulk RNA-Seq analysis. A two sided Wald test was used with Benjamini-Hochberg correction for multiple comparisons.

Supplementary Data 10: Summary of key marker genes for each retinal and other eye cell types used for cluster definitions in the single cell analyses included in this paper.
