# Supplementary material for: Single-cell analyses reveal transient retinal progenitor cells in the ciliary margin of developing human retina
Source: Nat Commun. 2024 Apr 26;15:3567. doi: 10.1038/s41467-024-47933-x (PMC11053058; doi:10.1038/s41467-024-47933-x)
Supplement: Supplementary file 14 — Reporting Summary [file 41467_2024_47933_MOESM14_ESM.pdf]

nature portfolio

Corresponding author(s): Majlinda Lako and Rachel Queen

Last updated by author(s): 21/03/2024

Reporting Summary

Nature Portfolio wishes to improve the reproducibility of the work that we publish. This form provides structure for consistency and transparency in reporting. For further information on Nature Portfolio policies, see our Editorial Policies and the Editorial Policy Checklist.

Statistics

For all statistical analyses, confirm that the following items are present in the figure legend, table legend, main text, or Methods section.

n/a	Confirmed	
☐	☒	The exact sample size (*n*) for each experimental group/condition, given as a discrete number and unit of measurement
☐	☒	A statement on whether measurements were taken from distinct samples or whether the same sample was measured repeatedly
☐	☒	The statistical test(s) used AND whether they are one- or two-sided<br>*Only common tests should be described solely by name; describe more complex techniques in the Methods section.*
☒	☐	A description of all covariates tested
☐	☒	A description of any assumptions or corrections, such as tests of normality and adjustment for multiple comparisons
☐	☒	A full description of the statistical parameters including central tendency (e.g. means) or other basic estimates (e.g. regression coefficient) AND variation (e.g. standard deviation) or associated estimates of uncertainty (e.g. confidence intervals)
☐	☒	For null hypothesis testing, the test statistic (e.g. *F*, *t*, *r*) with confidence intervals, effect sizes, degrees of freedom and *P* value noted<br>*Give P values as exact values whenever suitable.*
☒	☐	For Bayesian analysis, information on the choice of priors and Markov chain Monte Carlo settings
☒	☐	For hierarchical and complex designs, identification of the appropriate level for tests and full reporting of outcomes
☒	☐	Estimates of effect sizes (e.g. Cohen's *d*, Pearson's *r*), indicating how they were calculated

Our web collection on statistics for biologists contains articles on many of the points above.

Software and code

Policy information about availability of computer code

Data collection	No software used
Data analysis	10x Genomics Cellranger (Version 3.01)<br>Cellranger ATAC (Version 1.2), 10x Genomics<br>Spaceranger (Version 1.0)<br>DoubletFinder (Version 2.0.3), Bioconductor<br>Seurat (Version 4.3.0), https://satijalab.org/seurat/<br>Harmony (Version 0.1.1), Bioconductor<br>Monocle 3 (Version 1.3.1), Bioconductor<br>pycisTopic (Version 1.0.3), https://github.com/aertslab<br>pycistarget (Version 1.0.3), https://github.com/aertslab<br>scanpy (Version 1.9.5), https://github.com/scverse/scanpy<br>cell2Location (Version 0.13), https://github.com/BayraktarLab/cell2location<br>Spaniel (Version 1.12), Bioconductor<br>Bedtools merge (Version 2.30), Bedtools<br>Signac (Version 1.6), Bioconductor<br>ComplexHeatmap (Version 2.14), Bioconductor<br>Chromvar (Version 1.20), Bioconductor<br>SCENIC+ (Version 1.0.1), https://github.com/aertslab

For manuscripts utilizing custom algorithms or software that are central to the research but not yet described in published literature, software must be made available to editors and reviewers. We strongly encourage code deposition in a community repository (e.g. GitHub). See the Nature Portfolio guidelines for submitting code & software for further information.

Data

Policy information about availability of data

All manuscripts must include a data availability statement. This statement should provide the following information, where applicable:

- Accession codes, unique identifiers, or web links for publicly available datasets
- A description of any restrictions on data availability
- For clinical datasets or third party data, please ensure that the statement adheres to our policy

Data availability:
All the single cell data generated in this study have been deposited to GEO under the accession numbers: GSE234971 [GEO Browser - GEO - NCBI (beds.ac.uk)].

Code availability: The analyses scripts are described in: https://github.com/RachelQueen1/BBSRC_Retina 80. The code has been archived on Zenodo with the DOI [10.5281/zenodo.10599556].

Research involving human participants, their data, or biological material

Policy information about studies with human participants or human data. See also policy information about sex, gender (identity/presentation), and sexual orientation and race, ethnicity and racism.

Reporting on sex and gender	The study used female and male samples and detailed information about the sex of each sample is given in Supplementary Data 1 for scRNA-Seq data, Supplementary Data 3 for ST (spatial transcriptomics) data and Supplementary Data 5 for ScATAC-Seq data.
Reporting on race, ethnicity, or other socially relevant groupings	No such categorization was used in our analysis.
Population characteristics	Embryonic and fetal retina and eye samples were used for this study. Sex and developmental stage information is provided in Supplementary DATA 1, 3 and 5.
Recruitment	The human embryonic and fetal material was provided by Human Developmental Biology Resource (https://www.hdbr.org/).
Ethics oversight	Resource under ethics permission 23/NE/0135 issued by the NorthEast Newcastle and North Tyneside 1 Research Ethics Committee.

Note that full information on the approval of the study protocol must also be provided in the manuscript.

Field-specific reporting

Please select the one below that is the best fit for your research. If you are not sure, read the appropriate sections before making your selection.

☒ Life sciences ☐ Behavioural & social sciences ☐ Ecological, evolutionary & environmental sciences

For a reference copy of the document with all sections, see nature.com/documents/nr-reporting-summary-flat.pdf

Life sciences study design

All studies must disclose on these points even when the disclosure is negative.

Sample size	No sample size calculation was performed. Developmental stage was the most important criteria for this study which focuses on early retinal development. We could not dictate or control the acquisition of embryonic or fetal samples, hence for some developmental stages there were more one sample analysed.
Data exclusions	No data were excluded.
Replication	Due to the rarity of the samples we included replicates wherever possible. However we could not control the sample acquisition as this is dependent on pregnancy termination and consent procedures. When more than one sample per developmental stage was present, this was included in the analysis.
Randomization	N/A
Blinding	Blinding is not relevant to this study. This is a descriptive study and the experiments and analysis were designed to minimise risk of subjective interpretation.

Reporting for specific materials, systems and methods

We require information from authors about some types of materials, experimental systems and methods used in many studies. Here, indicate whether each material, system or method listed is relevant to your study. If you are not sure if a list item applies to your research, read the appropriate section before selecting a response.

Materials & experimental systems

n/a	Involved in the study
☐	☒ Antibodies
☒	☐ Eukaryotic cell lines
☒	☐ Palaeontology and archaeology
☒	☐ Animals and other organisms
☒	☐ Clinical data
☒	☐ Dual use research of concern
☒	☐ Plants

Methods

n/a	Involved in the study
☒	☐ ChIP-seq
☒	☐ Flow cytometry
☒	☐ MRI-based neuroimaging

Antibodies

Antibodies used

CRX, Abnova, H00001406-MO2 , Lot: KB191-4G11
Ki67, Abcam, ab15580 , Lot: GR3375617-1
Recoverin, Millipore-Merck, ab5585 , Lot: 3099956
RXRγ, Santa Cruz Biotechnology, sc-555 , Lot: D1013
RXRγ, Santa Cruz Bio-technology,sc-365252 , Lot: G1122
SNCG, Antibodies.com, A121664 , Lot: 25309
VSX2, Santa Cruz, sc-365519 , Lot: 000031213
Goat anti Mouse conjugated to Alexa488 (Jackson Immuno Research Laboratories, 115-545-146 , Lot: 157251
Donkey anti Mouse conjugated to Alexa488, Thermo Fisher, A21202 , Lot: 2266877
Donkey anti Rabbit conjugated to Alexa546, Thermo Fisher, A10040 , Lot: 1622582
Goat anti Rabbit conjugated to Cy3, Jackson Immuno Research Laboratories, 115-165-003 , Lot: 86185
Donkey anti Goat conjugated to Alexa 647, Thermo Fisher, A21447 , Lot: 2175459

Validation

All antibodies were purchased commercially and were validated by the manufacturer.

Plants

Seed stocks

Report on the source of all seed stocks or other plant material used. If applicable, state the seed stock centre and catalogue number. If plant specimens were collected from the field, describe the collection location, date and sampling procedures.

Novel plant genotypes

Describe the methods by which all novel plant genotypes were produced. This includes those generated by transgenic approaches, gene editing, chemical/radiation-based mutagenesis and hybridization. For transgenic lines, describe the transformation method, the number of independent lines analyzed and the generation upon which experiments were performed. For gene-edited lines, describe the editor used, the endogenous sequence targeted for editing, the targeting guide RNA sequence (if applicable) and how the editor was applied.

Authentication

Describe any authentication procedures for each seed stock used or novel genotype generated. Describe any experiments used to assess the effect of a mutation and, where applicable, how potential secondary effects (e.g. second site T-DNA insertions, mosiacism, off-target gene editing) were examined.
